# National survey among French adult nephrologists on red blood cell transfusion in the early post–kidney transplantation period: time for evidence-based guidance

**DOI:** 10.1093/ckj/sfag009

**Published:** 2026-01-13

**Authors:** Tanguy Sajous, Loic Lievre, Charlotte Colosio, Betoul Schvartz, Laetitia Mokri, Antoine Braconnier, Alain Wynckel, Philippe Rieu, Vincent Dupont

**Affiliations:** Department of Nephrology, University Hospital of Reims, Reims, France; Department of Nephrology, University Hospital of Reims, Reims, France; Department of Nephrology, University Hospital of Reims, Reims, France; Department of Nephrology, University Hospital of Reims, Reims, France; Department of Nephrology, University Hospital of Reims, Reims, France; Department of Nephrology, University Hospital of Reims, Reims, France; Department of Nephrology, University Hospital of Reims, Reims, France; Department of Nephrology, University Hospital of Reims, Reims, France; Department of Nephrology, University Hospital of Reims, Reims, France

To the Editor,

Anaemia is a common complication following kidney transplantation, with a reported prevalence ranging from 20% to 51%, depending on the haemoglobin (Hb) thresholds used [1]. Whatever the cause (low pretransplant Hb target, cytopenia-inducing immunosuppressive regimens, perioperative bleeding loss), early post-transplant anaemia has been associated with significantly higher risks of graft loss and all-cause mortality [1].

The role of red blood cell (RBC) transfusion in this context remains controversial, primarily due to concerns about HLA alloimmunization and the development of donor-specific antibodies (DSAs), which may compromise graft survival [2, 3]. Historically, transfusions were avoided in transplant recipients to reduce immunologic sensitization. However, more recent studies suggest that early post-transplant transfusion may not significantly affect the risk of alloimmunization, neither graft nor patient outcomes [4, 5]. Despite these emerging data, transfusion practices remain inconsistent, and no formal guidelines currently exist to direct transfusion decisions in the immediate post-transplant period.

To better understand current clinical approaches, we conducted a national cross-sectional survey of French nephrologists to assess transfusion practices during the first week after kidney transplantation. Anonymous data collection was conducted via a questionnaire administered through Skezia, a web-based tool designed for survey creation and distribution. The survey was distributed via national professional mailing lists and networks.

We received 104 responses from nephrologists working in 14 French cities (Table 1). Most were experienced clinicians: 38.4% had been practicing for 5–10 years, 25% for 10–20 years and 18.3% for >20 years. A total of 80.8% reported regular on-call responsibilities in nephrology. Nearly half (47.1%) of respondents identified kidney transplantation as their main clinical activity. Transplant centre volumes were substantial, with 38.5% of respondents working in centres performing >100 kidney transplants annually.

**Table 1:** Characteristics of the respondents.

Variables	Respondents (*N* = 104)
Position, *n* (%)	
Assistant/chief resident	22 (21.2)
Hospital practitioner	63 (60.5)
Associate/full professor	19 (18.3)
Experience (years), *n* (%)	
<5	19 (18.3)
5–10	40 (38.4)
10–20	26 (25.0)
>20	19 (18.3)
Main activity, *n* (%)	
General nephrology	28 (26.9)
Kidney transplantation	49 (47.1)
Dialysis	14 (13.5)
Nephrology intensive care	14 (13.5)
On-call duties	84 (80.8)
Transplants per year, *n* (%)	
<50	7 (6.7)
50–75	39 (37.5)
75–100	18 (17.3)
>100	40 (38.5)

Practice patterns showed substantial variability. A total of 41% of nephrologists reported avoiding transfusion unless absolutely necessary (e.g. in cases of active bleeding), while another 40% used transfusion more routinely but applied lower Hb thresholds than in the general population. Interestingly, 19% indicated that their transfusion decisions were not influenced by the transplant context. Alloimmunization risk remained a dominant concern, cited by 74% of respondents as the primary reason to avoid transfusion.

In a clinical vignette involving a stable 45-year-old post-transplant patient, the most cited transfusion threshold was Hb <70 g/l (54%), followed by Hb <65 g/l (21%). Factors prompting earlier transfusion included a history of coronary artery disease or visible bleeding, while younger recipient age (<30 years) was a reason to delay transfusion. When presented with a more complex clinical scenario, the cohort was nearly evenly divided: 52% chose not to transfuse while 48% would have done so. In exploratory comparative analyses between respondents who chose to transfuse and those who did not, transfusion refusal was significantly associated with professional position and declared transfusion attitudes: assistants and chief residents were more likely not to transfuse (*P* < .02), as were nephrologists, who reported using transfusion in <25% of kidney transplantations (*P* < .01) and those who declared a restrictive transfusion threshold of <7 g/dl (*P* < .01).

Our findings highlight significant heterogeneity in transfusion practices among French nephrologists, underscoring the absence of standardized guidance. While recent evidence suggests early transfusion may not increase immunologic risk [4, 5], concern over alloimmunization continues to drive clinical conservatism. These variations in practice likely reflect the complex interplay between individual clinician judgment, institutional protocols and evolving evidence.

To our knowledge, this is the first national survey to systematically assess transfusion attitudes in the early post–kidney transplant period in France. The results reveal a clear need for evidence-based, consensus-driven guidelines to harmonize transfusion practices and support clinical decision-making in this critical window of care.

## ACKNOWLEDGEMENTS

The authors want to thank the French Intensive Care Renal Network, Club des Jeunes Néphrologues and Groupe Spiesser for sharing the survey.

## Data Availability

No new data were generated or analysed in support of this research.
